# Myopathy due to carnitine palmitoyltransferase II deficiency: updating genetic aspects of the first publication in Brazil

**DOI:** 10.1055/s-0044-1779508

**Published:** 2024-02-23

**Authors:** Paulo José Lorenzoni, Cláudia Suemi Kamoi Kay, Renata Dal-Pra Ducci, Otto Jesus Hernandez Fustes, Paula Raquel do Vale Pascoal Rodrigues, Raquel Cristina Arndt, Rosana Herminia Scola, Lineu Cesar Werneck

**Affiliations:** 1 Universidade Federal do Paraná, Hospital de Clínicas, Departamento de Clínica Médica, Serviço de Neurologia, Serviço de Doenças Neuromusculares, Curitiba PR, Brazil.

**Keywords:** Muscular Diseases, Carnitine O-Palmitoyltransferase, Lipids, Genetics, Doenças Musculares, Carnitina O-Palmitoiltransferase, Lipídeos, Genética

## Abstract

Carnitine palmitoyltransferase II (CPT II) deficiency is an autosomal recessive inherited disorder related to lipid metabolism affecting skeletal muscle. The first cases of CPT II deficiency causing myopathy were reported in 1973. In 1983, Werneck et al published the first two Brazilian patients with myopathy due to CPT II deficiency, where the biochemical analysis confirmed deficient CPT activity in the muscle of both cases. Over the past 40 years since the pioneering publication, clinical phenotypes and genetic loci in the *CPT2* gene have been described, and pathogenic mechanisms have been better elucidated. Genetic analysis of one of the original cases disclosed compound heterozygous pathogenic variants (p.Ser113Leu/p.Pro50His) in the *CPT2* gene. Our report highlights the historical aspects of the first Brazilian publication of the myopathic form of CPT II deficiency and updates the genetic background of this pioneering publication.

## Resumo

Deficiência de carnitina palmitoiltransferase II (CPT II) é uma desordem de herança autossômica recessiva relacionada com o metabolismo do lipídio afetando músculo esquelético. Os primeiros dois casos de deficiência de CPT II causando miopatia foram relatados em 1973. Em 1983, Werneck et al. publicaram os primeiros pacientes brasileiros com miopatia por deficiência de CPT II, nos quais a análise bioquímica confirmou a atividade deficiente da CPT nos músculos em ambos os casos. Após 40 anos desde a publicação pioneira, fenótipos clínicos e *loci* genético no gene *CPT2* foram descritos, bem com os mecanismos patológicos foram melhor elucidados. A análise genética de um dos casos da publicação original apresentou variantes patogênicas em heterozigose composta (p.Ser113Leu/p.Pro50His) no gene *CPT2* . O nosso relato destaca os aspectos históricos da primeira publicação brasileira da forma miopática da deficiência de CPT II e atualiza as bases genéticas dessa publicação pioneira.

## INTRODUCTION

The carnitine palmitoyltransferase enzymes (CPTs) catalyse the transfer of long-chain fatty acids from the cytoplasm into mitochondria, where the oxidation of fatty acids takes place. 1 There are two sub-forms of CPT: CPT I, at the outer mitochondrial membrane, and CPT II, located in the inner membrane. 1

In 1973, DiMauro and DiMauro reported the first case of CPT II deficiency-causing myopathy in two brothers with recurrent myoglobinuria. 2 Since then, CPT II deficiency has been reported as one of the most common recessively inherited disorders of lipid metabolism affecting skeletal muscle by defects of long-chain fatty acid oxidation. 1 3 4 5 6 7 8

**Figure 1:**
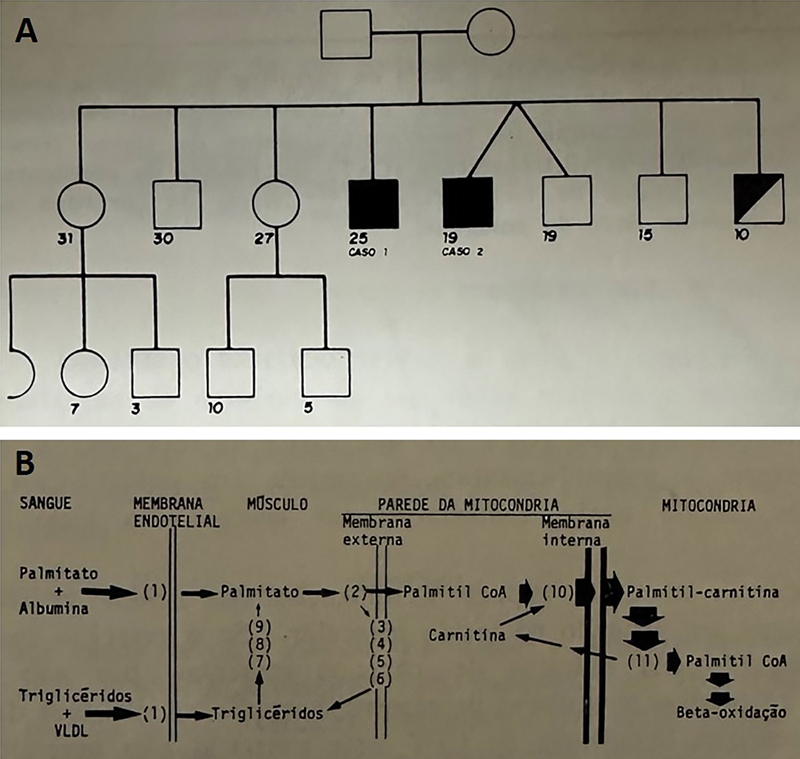
Original publication shows family pedigree (A: from original Figure 1 ) and metabolic pathway (B: from original Figure 4). [Portuguese language].

In 1983, Werneck et al. reported the first publication of myopathy due to CPT II deficiency in Brazil, describing two brothers ( Figure 1A ) aged 25 (‘case 1’) and 19 (‘case 2’) years, who presented muscle pain and decreased strength after prolonged exercise, which was made worse by colds. 9 At that time, one of the patients developed recurrent myoglobinuria and episodic renal failure; creatine kinase levels were normal between the crises but increased 100 times during myoglobinuria episodes; needle electromyography suggested denervation; and muscle biopsy showed increased lipid droplets by the ‘oil red O’ stain and increased activity of succinic dehydrogenase histochemical reaction.

In the 70s and 80s, the way to confirm CPT deficiency was by biochemical analysis of the muscle enzymatic activity, using a variety of biochemical methods that were not easily available. In the early years, one of the Brazilian authors had attended the ‘Houston Merritt Clinical Research Center for Muscular Dystrophy and Related Disorders’ of the Columbia University (New York, USA), which had expertise in muscle diseases of fatty acid metabolism. The proximity of Brazilian researchers to the leadership of this centre (Prof Salvatore DiMauro) allowed for the exchange of knowledge and, therefore, with the consent of patients, muscular enzymatic analysis was performed in New York. The biochemical analysis of muscle samples showed decreased carnitine-palmityl-transferase activity, with normal values for carnitine-octanoyl-transferase and carnitine-acetyl-transferase, confirming the deficiency of CPT in the muscle of ‘cases 1 and 2’ ( Table 1 ). Due to this contribution, one of the authors of the first worldwide publication (Prof Salvatore DiMauro) 2 also shares the authorship of the first Brazilian publication in this field. 9

**Table 1:** The enzymatic muscular activity described in the original article

Case	CPT*	COT*	CAT**	Carnitine***	
				Muscle	Serum
**1**	10,85	501,4	2,13	21,0	39,0
**2**	7,23	331,9	1,55	22,0	40,0
**Controls**	66,70 ± 7,3	235,0 ± 56,5	1,57 ± 0,48	19,3 ± 4,8	51,1 ± 11,1

Abbreviations: CAT, carnitine-acetyl-transferase; CPT, carnitine-palmitoyltransferase; COT, carnitine-octanoyl-transferase.

Notes: *pmoles of 14C-carnitin; **pmoles of acetylcarnitine; ***nmoles/mg.

The article published by Werneck et al. also discussed the metabolic pathway of fatty acid utilisation as an energy source for muscle during exercise in normal and pathological conditions with the knowledge of the time ( Figure 1B ). 9 Over the past 40 years since this publication, clinical phenotypes and genetic loci in the *CPT2* gene were described, and pathogenic mechanisms have been better elucidated. Three distinct phenotypes of the CPT II deficiency have been reported: a lethal neonatal form (hypoketotic hypoglycaemia and severe hepatomuscular symptoms); a severe infantile hepatocardiomuscular form (hypoketotic hypoglycaemia, liver failure, cardiomyopathy, and myopathy); and the classical myopathic form. 1 5 6 7

The myopathic form of CPT II deficiency (MIM #255110), also known as the ‘adult form’, is the most common presentation; it is relatively mild and considered benign, but difficult to diagnose. The patients reported by Werneck et al. had this phenotype. 9 This form presents most frequently in childhood or early adulthood and is usually characterised by attacks of myalgia, weakness, and/or rhabdomyolysis, precipitated mainly by exercise, but also by cold, fever, infection, or prolonged fasting. 1 3 6 8 In severe cases, rhabdomyolysis may lead to myoglobinuria and, consequently, renal failure. 1 5 6 However, persistent weakness is uncommon in patients with this phenotype. 1 3 There is a male predominance in CPT II deficiency, but the mechanism of this predominance is not clear. 1 The creatine kinase levels are markedly elevated during attacks, but generally remain within the reference range or are slightly elevated between attacks. 1 3 4 Although it is useful for differential diagnosis, muscle histological investigation in CPT II deficiency shows only unspecific myopathic changes with slight lipid accumulation. 3 4 In other words, the muscle histology cannot establish CPT II deficiency as there is no myopathological hallmark, in contrast with other muscle lipid disorders (i.e. carnitine deficiency). 3 4 In all patients, muscle CPT II deficiency must be confirmed biochemically or genetically. 3 The biochemical analysis must determine the CPT activity in muscle, as in the pioneering Brazilian publication, 1 and the magnitude of residual activity determines the severity, and tissue specificity, of the disease manifestations. 4 5 6 7 9 A definitive diagnosis requires the identification of mutations in the *CPT2* gene (OMIM*600650). Several mutations in the *CPT2* gene have been published with a correlation between genotype, metabolic dysfunction, and clinical presentation. 1 The phenotype of muscle CPT II deficiency might be influenced by the underlying mutation (about 60% of which are of missense type, 35% are of a null type and 5% are splicing mutations), which can allocate phenotypes into three subsets: ‘mild, severe and undefined’. 1 3 5 6 7 However, the biochemical consequences of the distinct mutations remain controversial.

In this publication, we update the genetic background of the original publication by Werneck et al in 1973. 9 In the original publication, genetic analysis of the *CPT2* gene was not available. However, ‘case 2’ is still attending medical appointments in the outpatient clinic of the Neuromuscular Centre at the ‘Hospital de Clínicas da Universidade Federal do Paraná’ (Curitiba, Brazil). At 60 years of age, he presents recurrent episodes of weakness and myoglobinuria after exercise, cold, or prolonged fasting (currently: one episode/month), as well as renal disease (glomerular filtration rate: 32.6 ml/min/1.73m ^2^ ) due to recurrent rhabdomyolysis and gout crisis. He has been taking L-carnitine 2g/day since infancy and, recently, some supplements (coenzyme Q10 100mg / C vitamin 400mg / NADH 10mg / L-valine 500mg / L-leucine 1g / L-isoleucine 500mg by day). His neurological examination is normal, except for the presence of bilateral ‘pes cavus’ (similar to at 19 years old). 9 Additionally, serum creatine kinase varied from 435 to 1754 U/L (Normal: <145 U/L) between the attacks; also, electrophysiological tests, such as needle electromyography and nerve conduction studies, were normal. The genetic analysis showed one heterozygous variant (p.Ser113Leu) in the *CPT2* gene by PCR/RFLP and compound heterozygosity for p.Ser113Leu/p.Pro50His in the *CPT2* gene by next-generation sequencing. The maternal and paternal allelic origin of these variants has not been determined.

More than 90 pathogenic variants have been identified in *CPT2* gene, the majority are predicted to produce amino acid substitutions or small deletions. Among Caucasian patients, there is a high frequency of a few mutations, especially p.Ser113Leu and p.Pro50His, which were found in our patient. 7 The p.Ser113Leu variant is the most common in patients with the myopathy form of CPT II deficiency, generally with allelic frequency of 60-70% and associated with ‘mild’ manifestations; this group, included patients with northern European and North American backgrounds. 3 4 5 6 7 The p.Pro50His mutation is also associated with a mild subset of CPT II deficiency and has been observed in about 6.5% of mutant alleles of patients with the myopathic form. 3 4 6 7

Despite these steps focused on identifying the diagnosis and management strategies of CPT II deficiency, this muscle disorder is still a mystery for many medical doctors, clinical geneticists, and even patients. In summary, our report highlights the historical aspects of the first publication of the myopathic form of CPT2 deficiency and updates the genetic background of the pioneering publication in Brazil.
